# Immune Protective Efficacy of China’s Traditional Inactivated and Attenuated Vaccines against the Prevalent Strains of *Pasteurella multocida* in Mice

**DOI:** 10.3390/vaccines9101155

**Published:** 2021-10-09

**Authors:** Li-Jun Guan, Ji-Jian Song, Yun Xue, Xia Ai, Zhi-Jun Liu, Li-Fang Si, Meng-Yun Li, Zhan-Qin Zhao

**Affiliations:** 1 Laboratory of Veterinary Biologics Engineering, College of Animal Science and Technology, Henan University of Science and Technology, Luoyang 471023, China; gljguanlijun@163.com (L.-J.G.); songjijian0702@163.com (J.-J.S.); xueyun6688@163.com (Y.X.); lzj_2132007@163.com (Z.-J.L.); slif2004@163.com (L.-F.S.); mengyun.li@163.com (M.-Y.L.); 2 Key-Disciplines Lab of Safety of Environment and Animal Product, College of Animal Science and Technology, Henan University of Science and Technology, Luoyang 471023, China; 3 Tianjin Key Laboratory of Agricultural Animal Breeding and Healthy Husbandry, College of Animal Science and Veterinary Medicine, Tianjin Agricultural University, Tianjin 300384, China; aixialucky@163.com

**Keywords:** *Pasteurella multocida*, traditional vaccines, mice, immune protection

## Abstract

Capsular type A and D strains of *Pasteurella multocida* are the main epidemic serogroups in pigs in China. In this study, we preliminarily evaluated the immune protective efficacy of the two traditional vaccines, an inactivated C44-1 aluminum-hydroxide-gel-adjuvanted (Alh–C44-1) vaccine and a live EO630 vaccine, against currently circulating strains of *P. multocida* in a mouse model. Mice immunized twice with conventional vaccines generated higher antibody titers, and significantly higher levels of IgG were observed in the mice inoculated with the inactivated Alh–C44-1 vaccine on day 35 (*p* < 0.05) than those with the live EO630 vaccine. The mice immune protection test showed that the vaccination groups had a 57% or 71% protection effect against the serogroup B strain, but had no protective effect against epidemic strains. In conclusion, our study found that the widely used traditional *P. multocida* vaccines in China provide good protection against homologous strains, but could not provide cross-protection against heterologous strains in a mouse model.

## 1. Introduction

*Pasteurella multocida,* a nonmotile, Gram-negative organism belonging to the genus *Pasteurella*, was first discovered by Perroncito in 1878. This bacterium was named after Louis Pasteur, who first recognized *P. multocida* as the causative agent of fowl disease in 1880 [1]. *P. multocida* is a widespread pathogen capable of infecting a wide variety of animals [2,3,4] and an opportunistic pathogen of humans. It is the primary causative agent of fowl cholera in chickens [5], hemorrhagic septicemia in ungulates, snuffles in rabbits [6], and progressive atrophic rhinitis or pneumonia in pigs [7]. Human infection with *P. multocida* typically occurs after the bites of cats or dogs, and can cause soft tissue infections and pulmonary disease [8,9,10].

There are five *P. multocida* capsular polysaccharide serogroups, based on capsular antigens with distinct structural and antigenic specificity, designated serogroups A, B, D, E, and F [11,12,13]. The prevalent serogroups of *P. multocida* may change over time in Chinese pigs. Prior to the 1990s, the dominant types of *P. multocida* were capsular serogroups A and B, but recent studies suggest that the most prevalent serogroups in China are capsular types A and D [14,15]. Pigs infected with *P. multocida* produce severe clinical symptoms, which cause considerable losses to the swine industry. Vaccination is currently considered the most effective approach to preventing this economically damaging infectious disease and controlling an infection at the population level [16].

Veterinary vaccines play a significant role in improving the health and welfare of companion animals and preventing animal-to-human transmission from both domestic animals and wildlife [17]. Developing a successful experimental vaccine involves the generation of a product that is available in the marketplace and can be used in the field to achieve the desired outcomes. The inactivated C44-1 aluminum-hydroxide-gel-adjuvanted (Alh–C44-1) vaccine and live EO630 vaccine are traditional vaccines for the prevention and control of pasteurellosis in China, which have been used for over 50 years [18,19,20]. While the conventional inactivated and live vaccines have a proven track record, little has been done to characterize their protective efficacy against the current prevalent *P. multocida* strains.

Therefore, in this study, for the first time, we study the protective efficacy of two *P. multocida* conventional vaccines, that have capsular serogroup B as their immunogenic antigen, against widely prevalent strains in a mouse model, to determine whether they can provide cross-serotype protection.

## 2. Materials and Methods

### 2.1. Bacterial Strains and Growth Conditions

The bacterial strains used in this study are *P. multocida* capsule serogroup A strains PM-5 [14] and PM-10 [14], and serogroup D strains PM-15 [21] and PM-G1, an isolate identified as a strongly virulent strain by our laboratory. Vaccine strain 44401 (C44-1) was purchased from the China Institute of Veterinary Drug Control (Beijing, China), and is a capsular serogroup B strain. Each sample was cultured on tryptic soy agar (TSA) containing 0.1% whole blood with lysed blood cells and 4% healthy calf serum for 12–15 h at 37 °C, as the F1 generation. It was then purified on a new TSA medium and cultured for 12 h at 37 °C as the F2 generation for subsequent research. The isolates were then treated with 15% skimmed milk powder and stored at −80 °C before further analysis.

### 2.2. Animals

All animal experiments in this study were performed in strict accordance with the recommendations delineated in the guidelines of the Animal Care and Use Committee of Henan University of Science and Technology (No. 20190516009). Female Kunming mice aged 6–8 weeks were purchased from the Animal Experiment Center of Zhengzhou University, China. These animals, five to seven per group, were kept in cages. The mice were provided with plenty of sterile water and diet, and after 3 days of acclimation, the experiment was commenced.

### 2.3. Live Vaccine and Adjuvant

The commercialized *P. multocida* live attenuated EO630 vaccine was purchased from China Animal Husbandry Industry Co., Ltd. (Lanzhou, China). The aluminum-hydroxide-gel adjuvant was purchased from the General Chemical Group (Parsippany, NJ, USA). The antigen of the EO630 live vaccine is capsule serogroup B. The bacterial content of the live vaccine was diluted to 5 × 10^7^ CFU/mL.

### 2.4. Inactivated Alh–C44-1 Vaccine Formulation

The inactivated Alh–C44-1 was prepared in strict accordance with the requirements of the Veterinary Biological Products Regulations of the People’s Republic of China, 2000 edition. Briefly, *P. multocida* C44-1 was cultured in trypticase soy broth (TSB) supplemented with 4% healthy calf serum and 0.1% whole blood with lysed blood cells, and incubated on an oscillator for 16 h at 37 °C. The mother liquor was then sub-cultured in a new liquid TSB medium with the same composition, in a ratio of 1:100, and was shaken and cultured for 16 h under the same conditions. The numbers of *P. multocida* cells were enumerated by plate counts at 12 h. The whole-cell bacteria were inactivated with 0.15% formalin and incubated for 48 h at 37 °C in an incubator. The inactivated cells were harvested by centrifugation at 4 °C, and resuspended with sterile phosphate-buffered saline (PBS). The inactivated vaccine was produced by emulsion with the Alh adjuvant. In the process of preparing the inactivated Alh–C44-1 vaccine, the ratio of Alh to C44-1 bacterial liquid was 1:5, and the final killed whole-cell vaccine (4 × 10^9^ killed cells/mL) was stored at 4 °C for further analysis.

### 2.5. Detection of Virulence

The virulence test of strain C44-1 (strain 44401 mentioned above) is described here, and exemplifies the method used for the other strains (PM-5, PM-10, PM-15, and PM-G1). A total of twenty-five Kunming mice were randomly assigned to five groups (A1-A5), and housed in a standard animal facility with ad libitum access to a normal rodent diet and water. The F2 generation colony of strain C44-1 was cultured in liquid TSB medium supplemented with 4% healthy calf serum and 0.1% whole blood with lysed blood cells, with incubation on an oscillator for 16 h at 37 °C. After identification with Gram-staining, the strain was sub-cultured at a ratio of 1:100 for 12 h. The numbers of *P. multocida* were enumerated with plate counts. The cultured C44-1 bacterial original fluid was serially diluted 10-fold in sterile phosphate-buffered saline (PBS). Then, cultures diluted 10-, 10^2^-, 10^3^-, and 10^4^-fold were generated. The mice in groups A1–A4 were inoculated intraperitoneally with 0.2 mL per mouse of the diluted cultures respectively, and the mice in group A5 were injected with 0.2 mL of sterile PBS, as a control group. A1–A5 groups of mice were isolated and kept for observation for 14 days, and the dead mice were dissected immediately to observe the gross pathology. The 50% lethal dose (LD_50_) was calculated on day 14 post-infection, as previously described [22].

### 2.6. Immunization and Challenge Schedule

One hundred and twenty-six Kunming mice were randomly divided into three groups, with forty-two mice in each group. The mice were subcutaneously injected (200 µL) on the back with the inactivated Alh–C44-1 vaccine, the live EO630 vaccine, or PBS alone as the negative control in week 0 and week 3. Mice in different vaccine groups were kept in different cages and isolated from each other. The immunized mice were then checked for any signs of adverse reactions or disease at 24 h post-vaccination. The antibody titers were measured in sera collected on days 0, 21, and 35. Blood was collected from the caudal vein of five mice from each of the vaccinated and PBS control groups, and the sera was stored at −80 °C for further analysis. For the challenge studies, mice (n = 5–7) from each group were intraperitoneally injected in week 5 with either a serogroup B, A, or D strain of a different challenge dose. The protective efficacy was determined by mice’s survival up to 14 days post-challenge. The mortality in the challenged mice was expressed in terms of the percentage survival and mean survival time (MST), and the percentage survival curve was plotted.

### 2.7. Measurement of Serum Antibody Titers

The serum of each mouse from different groups was assayed for the presence of antigen-specific immunoglobulin G (IgG) with an indirect enzyme-linked immunosorbent assay (ELISA). According to the checkerboard titration test, it was determined that the best antigen coating concentration is 1:8000, and the best serum dilution is 1:80. The supernatant of killed whole-cell C44-1 bacteria was collected by ultrasonication. Then, 96-well flat-bottomed microtiter plates were coated with the supernatant (1:8000) diluted in 0.05 M carbonate/bicarbonate buffer (pH 9.6, 100 µL/well), incubated for 1 h at 37 °C, and overnight at 4 °C. After the wells were washed and blocked, the serum antibodies diluted at 1:80 were added to the wells (100 µL/well) and incubated for 30 min at 37 °C. After washing the plates three times, 100 µL of horseradish peroxidase-conjugated goat anti-mouse IgG (Proteintech Group, Inc; Rosemont, IL, USA) diluted in blocking buffer (1:5000) was added to each well and the plates were incubated at 37 °C for 30 min, then the plates were washed five times. Next, 100 µL of TMB chromogenic solution was added to each well, protected from light for 10 min, and then 100 µL of TMB reaction stop solution was added to each well. Finally, the optical density value was measured by an ELISA reader at a wavelength of 450 nm.

### 2.8. Statistical Analysis

Antibody levels were analyzed statistically with one-way analysis of variance (ANOVA), and survival times were analyzed with the Kaplan–Meier method. For the statistical analyses, all data were expressed as means ± standard errors (SE). All statistical analyses were performed with the SPSS 16.0 software (SPSS Inc., Chicago, IL, USA), and graphics were produced with the Prism 8.01 software (Graph Pad, San Diego, CA, USA). The value of *p* < 0.05 was considered statistically significant.

## 3. Results

### 3.1. Virulence of the P. multocida Strains in Mice

To estimate the LD_50_ values of the test strains, 25 mice were used for each of the 5 isolates (125 mice in total). The cultures of the test strains were serially diluted. Mice in groups A1–A4 were intraperitoneally injected with 0.2 mL of culture diluted 10, 10^2^, 10^3^, and 10^4^ times respectively, and mice in group A5 were intraperitoneally injected with 0.2 mL of sterile PBS. The LD_50_ was calculated on day 14 post-infection by the Reed–Muench method [22]. The LD_50_ of the C44-1 vaccine strain was 4 CFU (Colony-Forming Units), those of the capsule serogroup A strains PM-5 and PM-10 were 11 and 26 CFU respectively, and those of the capsule serogroup D strains PM-15 and PM-G1 were 1.1 × 10^4^ and 3.4 × 10^3^ CFU, respectively. These results indicate that the virulence of *P. multocida* capsule serogroup A strains was greater than that of the capsule serogroup D strains. Mental disorder, hemorrhagic septicemia, and other symptoms were often observed in mice after infection. The dead mice were dissected and found to have swollen kidneys and spleen, as well as liver congestion.

### 3.2. Serum Antibody Titers

**Figure 1:**
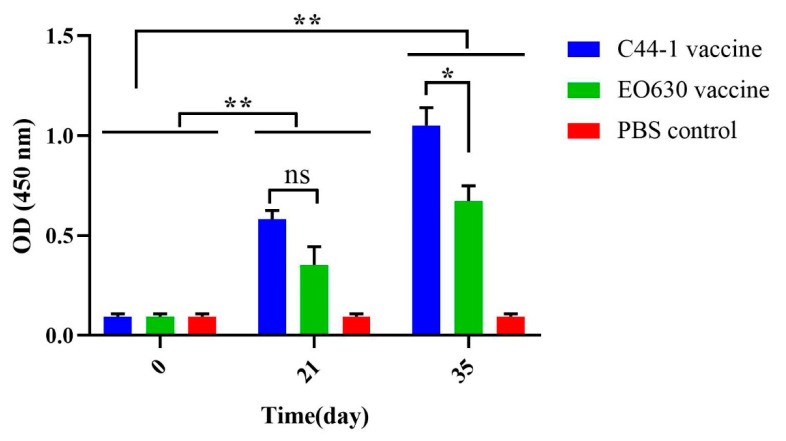
IgG antibody titers against *P. multocida* after vaccination. Blood samples were collected before each immunization (days 0 and 21) and before challenge (day 35). ‘C44-1 vaccine’ indicates mice inoculated with the inactivated Alh–C44-1 vaccine on days 21 and 35. ‘EO630 vaccine’ indicates mice inoculated with the live EO630 vaccine on days 21 and 35. ‘PBS control’ indicates mice inoculated with sterile PBS buffer on days 21 and 35. The *P. multocida*-specific antibody titers in the experimental and control groups were measured with an indirect ELISA and recorded at OD_450_. The error bars represent the SE. ** *p* < 0.01; * *p* < 0.05; ns, not significant.

The mouse serum antibody titers of the inactivated Alh–C44-1 vaccine group, the live E0630 vaccine group, and the PBS control group were measured with an ELISA before the primary vaccination, 21 days after the first immunization, and 14 days after the second immunization, as shown in Figure 1. Compared with the PBS control group, the mice immunized with the inactivated Alh–C44-1 vaccine or the live EO630 vaccine produced higher titers of specific IgG antibodies (*p* < 0.01) after the boost immunization, and the antibody titers increased to 1:256 and 1:128 on day 35, respectively. The serum antibody titers of the Alh–C44-1 vaccine group and the live EO630 vaccine group were also significantly different from that of the control group on day 21 (*p* < 0.01). Fourteen days after the second immunization, the antibody titer of the inactivated Alh–C44-1 vaccine group differed significantly from that of the EO630 group (*p* < 0.05), indicating that the immune response induced by the inactivated vaccine was stronger.

### 3.3. Protective Effect of Traditional Vaccines against P. multocida Serogroup B Strain

**Figure 2:**
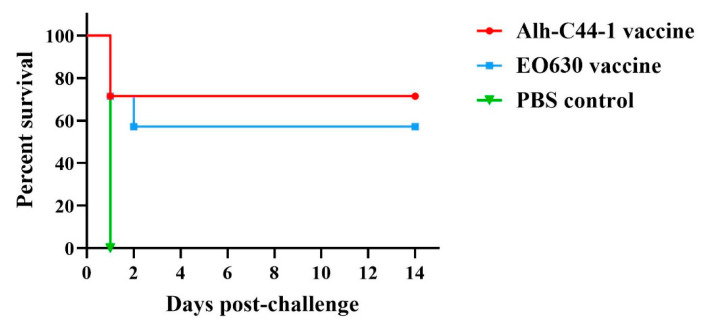
Survival rates of a mouse model immunized with traditional vaccines, after challenge with the serogroup B strain. All mice were challenged with a culture of virulent *P. multocida* C44-1 strain (12 LD_50_/mouse) by the intraperitoneal route. The mice were observed for 14 days post-challenge.

Twenty-one mice were vaccinated with the inactivated Alh–C44-1 vaccine, the live EO630 vaccine, or sterile PBS (Table 1), i.e., seven mice were randomly vaccinated twice with each vaccine, 21 days apart, and then challenged with 12 LD_50_ of the serogroup B strain, C44-1, in week 5. Strain C44-1 is a strong virulent isolate. The results of the challenge experiments are shown in Table 1. In the PBS control group, all the mice succumbed to infection, with 100% mortality. Five of the mice inoculated with the inactivated Alh–C44-1 vaccine survived, so the protection rate of the inactivated vaccine was 71%. Four of the mice inoculated with the live EO630 vaccine survived, so the protection rate of the live vaccine was 57% (Table 1). The percentage survival in the different groups of mice is shown in Figure 2. The mice immunized with PBS all died on the first day after the challenge. Immunization of the Kunming mice with the inactivated Alh–C44-1 vaccine clearly and significantly increased the survival time (10.29 ± 2.22; *p* < 0.01) relative to that of the control group, and the live EO630 vaccination group also showed a significantly prolonged survival period (8.57 ± 2.37; *p* < 0.01) relative to that of the PBS control. There was no significant difference between the two traditional vaccines.

### 3.4. Protective Effect of Traditional Vaccines against P. multocida Serogroup A Strains

**Figure 3:**
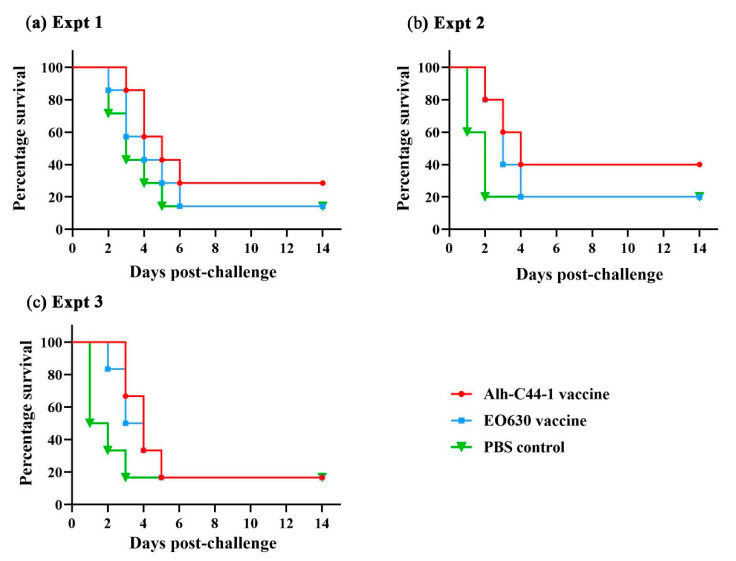
Survival rates of a mouse model immunized with traditional vaccines, after challenge with serogroup A strains. All mice were intraperitoneally challenged with (**a**) 18 LD_50_ of strain PM-5, (**b**) 29 LD_50_ of strain PM-5, or (**c**) 10 LD_50_ of strain PM-10. The mice were observed for 14 days after the challenge.

Mice were challenged with serogroup A strain PM-5 at two different doses, 18 LD_50_ and 29 LD_50_, and with 10 LD_50_ of strain PM-10, 14 days after the boost vaccination (Table 2). In Experiment 1, the challenge dose of strain PM-5 was 18 LD_50_, and 6/7 mice in the control group died, indicating that the experiment was valid (Table 2, Experiment 1). Two of the mice inoculated with the inactivated Alh–C44-1 vaccine survived, so the protection rate for the inactivated vaccine was 29%. One of the mice inoculated with the live EO630 vaccine survived, so the protection rate of the live vaccine was 14%. When the challenge dose was 29 LD_50_, 4/5 mice in the control group died, so the experiment was valid (Table 2, Experiment 2). The protection rate of the inactivated Alh–C44-1 vaccine in mice was 40% and that of the live EO630 vaccine was 20%. In Experiment 3, the challenge dose of strain PM-10 was 10 LD_50_, and 5/6 mice in the control group died (Table 2, Experiment 3). The survival rates after immunization with the inactivated Alh–C44-1 vaccine or the live EO630 vaccine were 17%, consistent with the result for the control group, indicating that the traditional vaccines induced no immune protection against strain PM-10. The percentage survival in the different groups of mice is shown in Figure 3. The MSTs of the inactivated Alh–C44-1 vaccine group and the live EO630 vaccine group did not differ significantly from those of the corresponding PBS control groups (*p* > 0.05) in Experiments 1, 2, or 3. Collectively, these data indicate that vaccination with the traditional vaccines did not induce significant protection against challenge with serogroup A strains of *P. multocida*, at either high or low challenge doses.

### 3.5. Protective Effect of Traditional Vaccines against P. multocida Serogroup D Strains

**Figure 4:**
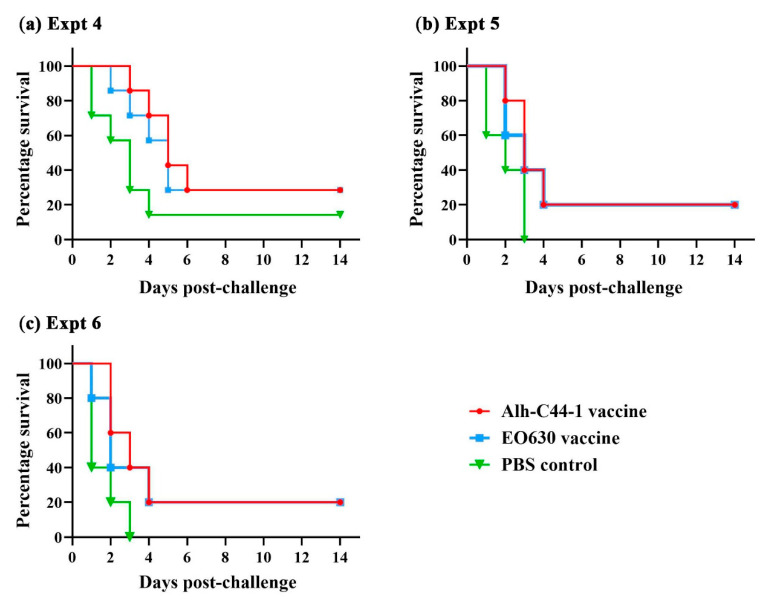
Survival rates of a mouse model immunized with traditional vaccines after challenge with serogroup D strains. All mice were intraperitoneally challenged with (**a**) 8 LD_50_ of strain PM-15, (**b**) 10 LD_50_ of strain PM-15, or (**c**) 25 LD_50_ of strain PM-10. The mice were observed for 14 days after the challenge.

Mice vaccinated with the inactivated Alh–C44-1 vaccine, the live EO630 vaccine, or sterile PBS were challenged with serogroup D strain PM-15 at two different doses, 8 LD_50_ or 10 LD_50_, or with 25 LD_50_ of strain PM-G1 at 14 days after the boost vaccination (Table 3). The results of the challenge experiments are shown in Table 3. When the challenge dose of the PM-15 strain was 8 LD_50_, 1/7 of the mice in the control group survived, and the experiment was valid (Experiment 4). Two of the mice inoculated with the inactivated Alh–C44-1 vaccine or the live EO630 vaccine survived, so the protection rates for the traditional vaccines were 29% in Experiment 4. When the challenge dose of strain PM-15 was 10 LD_50_, all the mice in the control group died, and the experiment was valid (Experiment 5). Only one of the mice inoculated with the inactivated Alh–C44-1 vaccine or the live EO630 vaccine survived, so the protection rates were 20% in Experiment 5. The remaining 15 mice were challenged with serogroup D strain PM-G1 (Table 3). When the challenge dose was 25 LD_50_, all the mice in the control group died. Only one of the mice inoculated with the inactivated Alh–C44-1 vaccine or the live EO630 vaccine survived, so the protection rates for these vaccines were 20% in Experiment 6. The percentage survival in the different groups of mice is shown in Figure 4. No significant difference was found between the inactivated Alh–C44-1 vaccine group (*p* > 0.05) or the live EO630 vaccine group (*p* > 0.05) and the PBS control group in Experiments 4, 5, or 6. Taken together, these data suggest that the traditional vaccines afford no protection against the current epidemic serogroup D strains, at either high or low challenge doses.

## 4. Discussion

In China, as in other parts of the world, *P. multocida* is a major causative agent of respiratory infection in farm and wild animals [23], causing the different symptoms of pasteurellosis, which range from minor local infection to fatal septicemia. It is a serious and highly contagious disease, affecting domesticated animals, especially buffalos [24], poultry [25], rabbits [26], and pigs. Vaccination is a successful method of controlling *P. multocida* infection [27], and inactivated vaccines, live attenuated vaccines, recombinant vaccines, subunit vaccines, and DNA vaccines against *P. multocida* have been extensively studied as preventive strategies [28,29,30]. For example, researchers studied various *P. multocida* vaccines providing protective immunity against progressive atrophic rhinitis in swine [16,31,32]. Traditional vaccines have played vital roles in controlling and eradicating infectious diseases for a long time [33]. Currently, the most widely used and marketed *P. multocida* vaccines in China are a whole-cell formalin-killed *P. multocida* C44-1 bacterin emulsified with aluminum-hydroxide-gel, and a live EO630 vaccine. These vaccines are widely used in China.

*P. multocida* is divided into five capsular serogroups of A, B, D, E, and F in terms of the capsular antigen [12,13], and both the C44-1 and EO630 strains are capsule serogroup B. In a previous study, we demonstrated that *P. multocida* capsular type A was the most prevalent serotype in China, followed by serogroup D [14], consistent with other researchers’ reports [7,34,35]. We have previously reviewed that the genes related to the capsule synthesis of *P. multocida* exist in the form of gene clusters [36]. Although the capsule synthesis genes of different serogroups of *P. multocida* are different, which could theoretically affect the cross-protection between different serogroups of strains, it still needs to be verified by experiments. Therefore, in this study, we used mice as a model to preliminarily evaluate whether the widely used conventional vaccines, the inactivated Alh–C44-1 *P. multocida* vaccine and the commercial live EO630 vaccine, in the Chinese market can provide immune protection against the current circulating strains.

The mice were subcutaneously immunized with two traditional inactivated vaccines twice, and then levels of serum IgG antibodies were determined by ELISA. A previous study [28] showed that mice vaccinated with a commercial alum-precipitated vaccine neither developed nor sustained sufficient levels of specific antibodies for a long period. However, in the present study, we showed that the vaccinated groups produced different levels of antibodies, and the highest antibody titers measured with an ELISA were observed on day 35. The antibody levels induced by the inactivated Alh–C44-1 vaccine differed significantly after the first and second immunization (*p* < 0.01), as did those induced by the live EO630 vaccine (*p* < 0.05). Therefore, the prime-boost immunization strategy is appropriate for these two vaccines. In general, the traditional vaccines can induce a humoral response [37]. This study only tested IgG levels induced by the two vaccines, and the humoral immune response is an important factor in resistance to *P. multocida* infection [38]. In order to evaluate the immune protection effect in depth, further studies are needed to test cellular responses such as induction of cytokine production (IFN-γ, IL-4, and IL-10, for example) in the natural host swine.

To evaluate the protective immunity of the vaccines, the vaccinated mice were challenged with virulent strains of *P. multocida* serogroups A, D, and B via an intraperitoneal route. Our data demonstrate that the traditional vaccines induced sufficient protection against challenge with the serogroup B strain, and the MSTs of vaccination groups were longer than the control group. However, the traditional vaccines did not induce significant protection against challenge with serogroup A or D strains, and there was no significant difference between the MSTs of vaccination groups and the control group. Although the inactivated Alh–C44-1 vaccine and live EO630 vaccine induced higher IgG levels, they did not afford protection against the currently prevalent virulent strains of serogroups A and D. These results showed that the traditional vaccine did not provide cross-protection against the virulent strains A or D, but had homologous protection against the virulent strain B, which may be related to the difference in the synthetic genes of the capsule antigen.

Our results are consistent with other studies suggesting that the cross-protection between different serotypes of *P. multocida* is weak. Shah et al. previously demonstrated that the *P. multocida* serotype B: 2.5 oil-adjuvant inactivated vaccine could provide cross-protection against serotype E: 2.5 in mouse immunological tests, but could not provide cross-protection against serotype B: 3.4 [39]. Abusalab et al. showed that the vaccine of the serogroup strain E was protective against challenge with the serogroup strain B, while the vaccine of B was not protective against E [40]. According to the R2 region of the *P. multocida* capsule gene cluster [11,36], it can be divided into classes I and II. Serogroups B and E belong to class II [41,42]. Theoretically, the two serogroups should protect each other, but the experimental results are opposite, so the cross-protection effect of *P. multocida* still needs to be verified by experiments. In addition, Wang et al. showed that the monovalent inactivated vaccine of the serogroup A strain had a protection rate against homologous strains in mouse experiments, but could not provide cross-protection against the serogroup B standard strain CVCC44701 [43]. Tao et al. showed that the protection rate of the prepared bovine serogroup A Pm12 inactivated vaccine was 80% against the type A strain and 60% against the type B strain in a mouse experiment [44]. The cross-protection between different serogroups of *P. multocida* is important in the prevention and control of this disease. Thus, it is still necessary to study the pathogenic mechanism of different serogroups of *P. multocida* and confirm the key factors of their interaction.

## 5. Conclusions

In conclusion, we preliminarily evaluated experimental vaccine efficacy of *P. multocida* serogroup B, inactivated C44-1 aluminum-hydroxide-gel-adjuvanted vaccine and live EO630 vaccine, in a mouse model in the present study. Our results showed that mice immunized with each of the two traditional vaccines produced a good immune response, and the two traditional vaccines provided adequate protection against homologous strains, but failed to provide cross-protection against heterologous strains, regardless of whether the challenge dose was high or low. Therefore, the polyvalent vaccine strategy should be considered to improve the efficacy of *P. multocida* traditional vaccines.

## Figures and Tables

**Table 1:** Immune protection afforded by traditional vaccines against *P. multocida* serogroup B strain.

Immunization Groups	Immune Dose (µL)	Challenge Strain	Challenge Dose (CFU)	No. of Mice	No. of Mice Survived	Protective Efficacy
Alh-C44-1 inactivated vaccine	200	C44-1	12 LD_50_	7	5	71%
EO630 live vaccine	200	C44-1	12 LD_50_	7	4	57%
Control	200	C44-1	12 LD_50_	7	0	0

**Table 2:** Immune protection afforded by traditional vaccines against *P. multocida* serogroup A strains.

Experiment	Immunization Group	Immune Dose (µL)	Challenge Strain	Challenge Dose (CFU)	No. of Mice	No. of Mice Survived	Protective Efficacy
Experiment 1	Alh-C44-1 inactivated vaccines	200	PM-5	18 LD_50_	7	2	29%
	EO630 live vaccine	200	PM-5	18 LD_50_	7	1	14%
	Control	200	PM-5	18 LD_50_	7	1	14%
Experiment 2	Alh-C44-1 inactivated vaccines	200	PM-5	29 LD_50_	5	2	40%
	EO630 live vaccine	200	PM-5	29 LD_50_	5	1	20%
	Control	200	PM-5	29 LD_50_	5	1	20%
Experiment 3	Alh-C44-1 inactivated vaccines	200	PM-10	10 LD_50_	6	1	17%
	EO630 live vaccine	200	PM-10	10 LD_50_	6	1	17%
	Control	200	PM-10	10 LD_50_	6	1	17%

**Table 3:** Immune protection afforded by traditional vaccines against *P. multocida* serogroup D strains.

Experiment	Immunization Group	Immune Dose (µL)	Challenge Strain	Challenge Dose (CFU)	No. of Mice	No. of Mice Survived	Protective Efficacy
Experiment 4	Alh-C44-1 inactivated vaccines	200	PM-15	8 LD_50_	7	2	29%
	EO630 live vaccine	200	PM-15	8 LD_50_	7	2	29%
	Control	200	PM-15	8 LD_50_	7	1	14%
Experiment 5	Alh-C44-1 inactivated vaccine	200	PM-15	10 LD_50_	5	1	20%
	EO630 live vaccine	200	PM-15	10 LD_50_	5	1	20%
	Control	200	PM-15	10 LD_50_	5	0	0
Experiment 6	Alh-C44-1 inactivated vaccine	200	PM-G1	25 LD_50_	5	1	20%
	EO630 live vaccine	200	PM-G1	25 LD_50_	5	1	20%
	Control	200	PM-G1	25 LD_50_	5	0	0

## Data Availability

All data generated are contained in the present manuscript.
